# Are Burned Babies and Mass Graves a Global Health Crisis? What Does Decolonization Got to Do With It? Comment on "The Rhetoric of Decolonizing Global Health Fails to Address the Reality of Settler Colonialism: Gaza as a Case in Point?"

**DOI:** 10.34172/ijhpm.8661

**Published:** 2024-12-10

**Authors:** Anonymous

**Affiliations:** Palestinian doctor.

**Keywords:** Genocide, Settler Colonialism, Palestine, Gaza

## Abstract

In this commentary, the author situates the article of Engebretsen and Baker in the larger history of exceptionalizing and evading Palestine in the global health literature. The political root causes for ill health in Palestine such as settler colonization, apartheid and racism are evaded and deemed "too political" and Palestinian health is flattened to the humanitarian realm, thus reinforcing Palestinian dependency on humanitarian aid, rather than subjects of Israeli necropolitics. The commentary focuses on this moment of genocide in Gaza and the importance of shifting the narrative on Palestinian health and global health more generally to focus on current imperialism, wars and settler colonialism. The commentary highlights the importance of showing moral clarity at this moment and center the Gaza Genocide in classrooms, publications and conferences rather than avoiding "controversy" and developing a moral outrage when it is no longer useful and after the smell of death has dissipated.

 Volumes have been written on the critique of global health,^1,2^ a field that claims to strive for health equity and justice across the globe, yet remains captive to the existing power structures where scholars in Ivy league universitas, grants and research institutes in the global north dictate research agendas and produce knowledge about diseases debilitating the lives of some of the poorest peoples in the global south and debating technical interventions,^3^ often packaged as “culturally sensitive.” With endless publications on infectious and non-communicable diseases in southern countries, global health experts rarely address the infrastructure and architecture of global misery and the upstream drivers that have created and maintain poverty, de-development, humiliation and subjugation, and the conditions producing ill health for the wretched majority of the inhabitant of this planet.^4,5^ As Paul Farmer and colleagues argue in their book “*Reimagining Global Health*,” a myopia exists about framing issues of causality and responses when thinking of health equity.^6^ This commentary points the attention to another “myopia” concerning the current imperial and settler colonial wars, specifically the genocide in Gaza.

 For those of us living and working with our communities in southern countries, we ambivalently watched the growing empire of global health and saw the birth and development of an ecosystem of academics, journals, editors, non-governmental organizations, and funders. This eco system carries its own contradictions and is encumbered by the conditions that birthed it. While global health strives for health equity and justice, noble ideals that require commitment to human dignity, sovereignty, decolonization, redistribution of wealth, demilitarization and reparations, the field is well guarded by its gate keepers of funders, editors and governments who are much less keen on creating controversies and challenging states, economic and military institutions that are the perpetrators of much of the harm causing health inequity. The result is a performative display of social justice seeking landscape that is tuned down, cautious and rarely transformative. These contradictions are most notable in the Palestinian context as the gap between the extreme cautiousness and censorship of the articles and reports on one hand, and the brutality of life under colonization on the other hand, is flagrant. Thus, work in Palestine offers an opportunity for global health experts to enrich their CV with “conflict zone expertise,” humanitarian missions, and publications that focus on “cultural” patterns of behaviors, statistics on post-traumatic stress disorder and attacks on healthcare, without mentioning or challenging Israeli settler colonization. Wars, death and disability in colonized Palestine, and in the global south more generally, has become a playground for theory and praxis^7^ for academics and practitioners to enrich their portfolios, while being careful not to harm their future employment options by pointing a finger to the perpetrators and enablers of diseases, disability or death.

 When a movement calling to decolonize global health grew and developed, it was largely coopted by liberal reforms and distracted with questions of representation, language, curriculum and some attention to partnership with global south scholars.^1,8,9^ While these issues are important, the movement has failed to address the core infrastructure and structures that engineer and drive ill health in the world.^9-11^ Colonialism is taught as a historical event with vague traces in our lived reality. Current imperial wars and weapon industries that kill millions globally, ravish countries, societies and the environment are rarely addressed, or even avoided, in these discussions.^12^ Global capitalism with its neocolonial architecture, fortified with trade agreements to maintain and reproduce debt^13^ is not connected to discussion on class and poverty.^5,14^ Extractive capitalism turning nature into natural resources to circulate in global markets, shaping poverty, exploitative labor and ecocide are evaded in discussions on planetary health. There is very little grasp of how the colonial order of things metamorphosed into neo colonialism and imperialism. Schools of global health do not teach students that the global north, with its wealth and surplus, is a product of the colonized world just as much as the global south with its poverty, wars and debts are a product of colonization.^4,15^ In classes, students are not taught how most significant decolonization movements in the south were crushed to death by governments in the north to prevent true sovereignty over power and wealth.^14,16^ As Rabea Eghbariah noted in international law, “Legal scholars tend to sharpen their pens after the smell of death has dissipated and moral clarity is no longer urgent.” We can observe a similar dynamic in global health where scholars avoid tackling contemporary imperial wars (Like the war on Iraq, Afghanistan, Yemen, and Palestine) where moral clarity is urgently needed and prefer to focus on sites of former colonial wars.

 For anyone genuinely interested in all those debates, and for those passionate about social, political and environmental justice, the Question of Palestine^17^ represents a natural moral compass.^18,19^ A place for scholarship,^20^ praxis^7^ and a litmus test.^21^ It is a site of moral clarity that encapsulates the dynamics between the global north and the global south on a small stretch of land and over a condensed epoch. In the wake of decolonization, when most nations in the world were gaining independence and expelling foreign colonizing armies, Palestine was colonized.^22^ A European settler colonial movement, supported by the empires of the time, conducted a swift land theft in the middle of the twentieth century.^23^ Every tool in the textbook of settler colonization was practiced in Palestine. Eliminating the native^24^ took the shape of racialization, securitization, expulsion, and minoritization.^25^ A new lexicon and logic were created and enforced with fire, steel and a divine decree. The land of Palestine became Israel, a Jewish state for the Jewish people.^26^ Palestinians were racialized as non-Jews and thus a demographic and security threat simply by being non-Jews.^27^ People inhabiting their land for centuries became an obstacle in the face of the colonization of their land and thus deemed dangerous and in need of dispossession. If they resist their dispossession or try to return to their homes, they become “terrorists” or “trespassers,” a justified target for killing.^28^ Palestinians were not allowed to return to their homeland due to the same reason they were expelled, for being non-Jews and an obstacle to a Jewish majority in a newly created ethno-nation state: a singularly Jewish State. What was puzzling to Palestinians was not only the seamless acceptance of this new reality (in the name of the horrors of Jewish racialization and genocide in Europe), but the celebration of Israel as a post war success story, a new democracy blooming the dessert. Israel subjected the Palestinians to every possible colonial tactic^29^ including killing, intoxication,^30^ exploitation of labor,^31^ humiliation, traumatization,^32^ theft of resources,^33^ apartheid,^34^ and most recently genocide. Palestinians were deemed to live under a machinery of sadistic bureaucratic brutality^35^ that controls their movement and access to land and resources. Israeli bureaucrats and soldiers could determine the time Palestinians spend on humiliating checkpoints, or the amount of paperwork needed to apply for medical permit. Young soldiers and illegal Israeli settlers on Palestinian land spend their early twenties brutalizing an entire nation, shooting water tanks, maiming and blinding protesters or cutting lives short with impunity,^35^ before continuing their academic and career path.

 Rather than Palestine taking a central stage in the global health landscape, situating it along other sites of necropolitics like the plantation, township, reservation, ghetto or concentration camp, showcasing in real time the toxic health effects of land alienation, codified racism, traumatization, impoverishment, ghettoization, mass incarceration, torture, killing and endless other modes of oppression, and most importantly having global health scholars and advocates call out these forces and work towards their abolition, global health schools and literature have chosen to exclude Palestinian health from discussion on racism, colonialism, apartheid or indigenous health. As we have mentioned earlier, global health scholars, like legal scholars, try not to challenge the zeitgeist and “sharpen their pens after the smell of death has dissipated and moral clarity is no longer urgent.”^36^ As several scholars have observed recently, during colonialism and enslavement, medical institutions and journal adopted the position of the dominant colonizing power and were complicit in producing and reproducing pseudoscience about the “inferiority of the colonized,” “madness of escaped enslaved people”^11^ or in silence as in the case of many medical journals during the holocaust.^37^ When it comes to Gaza and Palestine more generally, we see the same pattern of silence and complicity by the global health industry. A small and well demarcated space is allocated for Palestinian health in discussing refugees, minority and conflict health^[1]^.^38^ Even in those places, to publish or be given a stage, Palestinians have to castrate their language and terminology, flatten their anger and walk between the linguistic landmines to describe their reality in order not to be rejected by reviewers and funders.

 The global health industry reduced Palestinians to their bare biological needs, focusing on delivery of food, medications and access to healthcare, protecting them from dying from hunger or diseases while not tackling the settler colonization ravishing their lives and environment.^44^ It has kept Palestinians trapped in a humanitarian condition,^45^ always in need of humanitarian support by a class of saviors.^46^ In the same manner, scholarship on Palestinian health was reduced to that humanitarian realm, assessing needs and intervention on small, scattered projects while avoiding tackling the root causes^44^ preventing Palestinians from living in dignity. Attempts to call out the root causes as drivers of Palestinian ill health were deemed too political and controversial by most global health journals and organizations.^44^

 The decolonizing global health movement did not even touch issues related to Palestine because to most global health scholars, Palestine is not a colonial question. It is a question of armed conflict that is better avoided.

 The genocide in the Gaza Strip occurred against the backdrop of all the above-mentioned dynamics. Global health scholars, journals and institutions, who for decade have been avoiding an honest discussion on one of the most prevailing colonial questions of our time and the most inflammatory settler colonial frontier, were faced with witnessing a genocide televised right into their phone screens. Decades of global health acceptance and adaptation to an ever more heinous status quo as Palestinians in general and Gazans in particular were brutalized^47^ and told to accept this reality, have paved the way to this very moment where a genocide is evaded in global health. Palestinians were censored and largely prevented from connecting those drivers to their ill health. Even when the Israeli medical establishment took an active role in the workings of apartheid and settler colonialism by colluding completely with state and military apparatus, participating in torture of prisoners^48^ and building a medical faculty in a settlement,^49^ it was not held accountable or sanctioned. So, have we reached a point where we can connect an image of a beheaded baby in Rafah^50^ to an Israeli artificial intelligence assisted^51^ genocide with American Bombs or is that too political for global health? Do we need to produce rigorous scientific papers connecting bombing a displaced population to excess mortality and starvation to low birth weight, with statistical significance so we can prove that genocide is bad for health?

 With every passing day we witnessed hospitals flattened,^52^ doctors killed and tortured, entire families erased, children burnt, amputated or buried in mass graves.^53,54^ To all those shocking atrocities caused by an industrialized killing machine, global health bodies chose not only silence and active unseeing, but a suppression of free speech, censorship, and attacks on health workers mobilizing in solidarity to end the genocide.^37^ Decades of Israeli impunity, mirrored with impunity in the global health literature, where addressing every human right abuse and violation was deemed too political and evaded, led to this moment where a genocide is evaded. A genocide of such a scale that should be occupying central stage in global health journals and conferences, in World Health Organization (WHO) statements and advocacy, was reduced to a minimum of shallow discussions about the humanitarian crisis and a need for intervention at best, while other scholars and journals were justifying the genocide including the attacks on hospitals for military purposes.^55^

 The article of Engebretsen and Baker,^56^ together with few other articles^37,57-59^ by brave scholars, situating Palestine in the heart of global health debates, demanding that individuals and institutions take responsibility and a position on a live streamed genocide, herald a shift in writing about Palestine in the health literature. This shift, bolstered by a growing student movement around the globe calling out Israel as a criminal perpetrator and challenging the silence and collusion of medical institutions that have built and maintained the infrastructure of Israeli impunity and Palestinian dehumanization and is allowing a genocide to unfold, represents our hope for a better future where global health as a discipline can be reclaimed by the people of conscience to actually respond to the pressing questions of our times. It took decades for medical journals to confront their silence and complicity with the crimes of the holocaust, colonialism, segregation in the US south and apartheid South Africa.^37^ Palestine is knocking on the sides of the tank^60^ and demanding a new world order where we can envision a future without racial supremacy, settler colonialism and apartheid.

 At this dark moment, when so many of us feel hopeless and helpless, it is important to remember that a new world is possible. If all this machinery of global violence, keeping millions homeless and incarcerated in the US and massacring Palestinians in Gaza, is not only imaginable but is a lived reality, a new world with much less suffering and injustice is not only imaginable but necessary and possible. The medical institution is a wealthy and powerful institution that has enormous power to center and fight a genocide as an issue of global health justice. We are guided by the words of bell hooks that “the classroom remains the most radical space of possibility in the academy.” We have the duty to center Palestine and Gaza in our classrooms, journals, conferences, and statements. The current silencing around Palestine and Gaza in global health is change to enabling the genocide to unfold smoothly and it is time to start a movement halting the wheels of colonial violence by exposing, isolating and delegitimatizing the perpetrators and enablers of the Gaza Genocide.

## Ethical issues

 Not applicable.

## Conflicts of interest

 The author declares that he/she has no conflicts of interest.

## Disclaimer

 The irony of writing anonymously in a commentary about courage and silencing is not lost upon us. As Audre Lorde teaches us, we are not always blessed with choosing the time and place of our battles. I battle from the belly of the beast, torn between moral duty and safety and pragmatic considerations.

## Endnotes

 [1] For more on the exclusion of Palestine from indigenous health see Asi et al^38^ and Tanous^39^ and for the silencing in medical journal see Wispelwey et al.^40^ This exclusion mirrors the exclusion of Palestinian trauma from the trauma literature and social suffering as detailed by Sayigh.^41^ For the exclusion of Palestine from progressive political discussions in general see Hill and Plitnick^42^ and the seminal work of Said.^43^

## Editor note

 According to the Committee on Publication Ethics (COPE), anonymizing authors is permissible under special circumstances, such as if publishing their names would compromise their safety. Accordingly, we published this paper anonymously at the author’s request. The journal has confirmed the author’s legal identity, and we followed the usual editorial processes without any conflicts of interest. Authorship forms were signed by the author using his/her legal name. This information is stored in the journal management system, and only the editorial team has access to it.
